# Adenosine Signaling as a Central Integrative Network in Cellular Stress Responses and a Therapeutically Actionable Target in Human Disease

**DOI:** 10.3390/biom16050732

**Published:** 2026-05-16

**Authors:** Shakta Mani Satyam, Mohamed El-Tanani, Wasim Iyad Alghoul, Malak Moones Abedi, Shabil Fathah Farook, Ibrahim Khalil Alabid, Mohammed Dalbah, Natasha Nasser, Samreen Fazal, Mariam Radhi Al-Talqani, Mohammed Mahmood Ali, Ebrahim Safaii, Wed Burhan Jameel Al-Shammari, Burhanuddin Murtaza Patanwala

**Affiliations:** 1 Department of Pharmacology, RAK College of Medical Sciences, RAK Medical and Health Sciences University, Ras Al Khaimah 11172, United Arab Emirates; 2 RAK College of Pharmacy, RAK Medical and Health Sciences University, Ras Al Khaimah 11172, United Arab Emirates; 3 Saqr Hospital, Emirates Health Services, Ras Al Khaimah 11172, United Arab Emirates

**Keywords:** adenosine signaling, CD39–CD73 axis, adenosine receptors, metabolic stress signaling, tumor microenvironment, immune modulation, hypoxia–HIF-1α pathway, translational therapeutics

## Abstract

Adenosine has emerged as a central metabolic signal linking cellular stress to systemic physiological adaptation. Under conditions such as hypoxia, ischemia, inflammation, and tissue injury, extracellular adenosine triphosphate (eATP) released from stressed cells is sequentially metabolized by the ectonucleotidases CD39 and CD73, generating adenosine that accumulates in the extracellular microenvironment. This stress-responsive nucleoside activates four G-protein-coupled receptors (A1, A2A, A2B, and A3), triggering intracellular signaling networks including the cyclic adenosine monophosphate–protein kinase A (cAMP–PKA), mitogen-activated protein kinase (MAPK), phosphoinositide 3-kinase–protein kinase B (PI3K–Akt), and hypoxia-inducible factor-1 alpha (HIF-1α) pathways. Through these integrated mechanisms, adenosine orchestrates diverse physiological processes such as vascular regulation, metabolic adaptation, immune modulation, and cellular survival. In the cardiovascular system, adenosine promotes coronary vasodilation and ischemic preconditioning, limiting reperfusion injury. In pulmonary tissues, it mediates acute anti-inflammatory responses but may also drive chronic fibrotic remodeling. Within the central nervous system, adenosine functions as a neuromodulator regulating neuronal excitability, sleep–wake homeostasis, and neuroprotection. In the tumor microenvironment, hypoxia-driven adenosine accumulation suppresses cytotoxic T cell and natural killer activity, facilitating immune evasion and tumor progression. Collectively, adenosine signaling represents a central integrative network that links metabolic stress sensing to coordinated cellular adaptation while simultaneously emerging as a clinically actionable therapeutic target across cardiovascular, inflammatory, neurological, and oncological diseases.

## 1. Introduction

Biological survival relies on the ability of living organisms to sense environmental and metabolic stress and to coordinate rapid protective responses across multiple tissues and organ systems. Metabolic stress refers to a condition in which cellular energy demand exceeds supply, resulting in disruption of adenosine triphosphate (ATP) homeostasis, redox imbalance, and activation of adaptive signaling pathways. Among the molecular systems that translate metabolic stress into coordinated physiological responses, adenosine occupies a uniquely central position, functioning as a dynamic metabolic and immunoregulatory signal [1]. Adenosine is a purine nucleoside generated primarily through the breakdown of ATP, a central intracellular energy molecule that also functions as an important extracellular signaling mediator [2,3]. Under homeostatic conditions, intracellular ATP concentrations remain high, while extracellular nucleotide levels are strictly regulated [4,5]. This tight regulation prevents inadvertent activation of purinergic signaling pathways, ensuring that adenosine functions as a precise indicator of cellular stress rather than a constitutive signaling molecule [6,7]. In this context, the concept of cellular adaptation and recovery capacity is defined as the ability of cells and tissues to dynamically respond to, withstand, and restore homeostasis under metabolic, inflammatory, or hypoxic stress conditions. Adenosine signaling contributes centrally to these processes by coordinating energy balance, immune modulation, and cytoprotective responses, thereby enabling sustained cellular and tissue function under adverse conditions.

Under physiological conditions, low extracellular concentrations of adenosine act as a baseline signaling molecule that helps maintain homeostasis across multiple systems [8]. In contrast, during conditions such as hypoxia, inflammation, or tissue injury, extracellular adenosine levels increase and function as a metabolic stress signal that initiates adaptive and protective responses [9,10]. Similarly, extracellular ATP accumulates in various pathological states, including ischemia, hypoxia, infection, inflammation, physical injury, and metabolic stress, where it contributes to stress signaling and modulation of cellular responses [11]. The rapid formation of extracellular ATP is terminated by a cascade of ectonucleotidases, which degrade accumulated ATP and adenosine diphosphate (ADP) into adenosine [12,13]. A cluster of differentiation 39 (CD39; ENTPD1: Ectonucleoside triphosphate diphosphohydrolase-1) sequentially hydrolyzes extracellular ATP and ADP to adenosine monophosphate (AMP), while a cluster of differentiation 73 (CD73; NT5E: Ecto-5′-nucleotidase) catalyzes the conversion of AMP to adenosine, completing the extracellular ATP-adenosine signaling cascade [14]. Extracellular nucleotide metabolism involves both canonical CD39–CD73-mediated hydrolysis of ATP to adenosine and non-canonical pathways, including CD38/CD203a/CD73-mediated NAD^+^ metabolism and extracellular cAMP–adenosine conversion, thereby providing multiple convergent routes for adenosine generation under stress conditions [14,15,16]. The production of extracellular adenosine functions as a biochemical alarm signal, conveying information about cellular distress to surrounding cells and distant tissues. Unlike the majority of signaling molecules being relevant to specific physiological contexts, adenosine functions as a global metabolic stress signal that elicits comprehensive physiological responses in several organ systems [17]. These pathways collectively demonstrate that cellular stress adaptation mechanisms converge across multiple disease states, including cancer, cardiovascular disorders, and metabolic dysfunction, reflecting shared underlying regulatory networks rather than isolated disease-specific processes. Recent experimental and translational studies underscore the pivotal role of metabolic stress–responsive signaling networks across a wide spectrum of pathological conditions. These include tumor progression and cancer stem cell biology, cardiometabolic disease modeling, chemotherapy-induced organ toxicity, wound healing, ocular metabolic disorders such as diabetic cataract, and lipid metabolism dysregulation. Collectively, these findings indicate that cellular stress adaptation pathways serve as shared mechanistic frameworks that interconnect oncological, cardiovascular, and metabolic diseases [18,19,20,21,22,23,24,25]. Upon release, the newly synthesized adenosine acts on all its four receptor subtypes (A1, A2A, A2B and A3) that are members of the large family of G-protein-coupled receptors (GPCRs) expressed on different types of cells in various organs [26]. Upon GPCR binding by adenosine, distinct downstream signaling pathways are activated that, in turn, modulate vascular tone, immunity, energy homeostasis, neural activity, and cellular proliferation [27,28,29].

Historically, the biological significance of adenosine was first identified within the cardiovascular system [30]. Early studies demonstrated that adenosine slows atrioventricular conduction, induces coronary vasodilation, and protects myocardial tissue during ischemic injury [31,32,33,34]. These discoveries led to the clinical implementation of adenosine in arrhythmia management and diagnostic cardiac stress testing. However, over the past two decades, advances in molecular pharmacology, immunology, and tumor biology have significantly expanded our understanding of adenosine signaling beyond the cardiovascular system [35,36]. Current evidence demonstrates that adenosine participates in a broad spectrum of physiological and pathological processes including immune regulation, airway inflammation, neuronal modulation, tissue remodeling, and tumor immune evasion [1,37,38].

This expanding knowledge has transformed the adenosine system from a niche cardiovascular signaling pathway into a central regulatory network with profound implications for human health and disease. Complementary clinical and translational evidence also suggests that therapeutic modulation of metabolic and signaling pathways through lifestyle interventions, RNA-based lipid-lowering strategies, chronopharmacology-guided drug delivery, and targeted anticancer therapies may significantly influence disease progression and long-term outcomes, reinforcing the importance of integrative precision medicine approaches in cardiometabolic and oncologic care [25,39,40,41]. Adenosine signaling has now emerged as a major therapeutic target across multiple clinical domains, including cardiology, pulmonology, neurology, and oncology. Importantly, the ability of adenosine signaling to coordinate rapid adaptive responses and facilitate recovery following cellular injury highlights its role in maintaining functional stability across tissues, particularly under conditions of repeated or chronic stress exposure. The diversity of adenosine-mediated effects is largely attributable to the differential expression of its receptors, receptor-specific coupling to intracellular G-proteins, and the tissue-specific activation of downstream signaling networks. The spatial and temporal dynamics of extracellular adenosine production, combined with receptor heterogeneity, enable fine-tuned modulation of both acute and chronic stress responses [42,43].

Adenosine signaling integrates with several major intracellular pathways to mediate cellular adaptation. Activation of A2A and A2B receptors stimulates cyclic adenosine monophosphate (cAMP) production, leading to activation of protein kinase A (PKA) and subsequent regulation of transcription factors such as Cyclic AMP Response Element-Binding Protein (CREB) [44,45]. Through this pathway, adenosine influences metabolic adaptation, inflammatory gene expression, and cell survival. Parallel engagement of the mitogen-activated protein kinase (MAPK) pathway regulates cell proliferation, differentiation, fibroblast activation, and cytokine production, particularly under inflammatory or fibrotic conditions [46,47]. The phosphatidylinositol-3-kinase (PI3K)–Akt axis further mediates anti-apoptotic signaling and promotes tissue resilience under metabolic or oxidative stress [48,49]. Additionally, hypoxia-inducible factor-1α (HIF-1α) amplifies adenosine signaling during oxygen deprivation by upregulating CD39 and CD73 expression and increasing receptor sensitivity, thereby establishing a feed-forward loop that enhances tissue adaptation to hypoxic stress [50,51,52]. These integrated signaling networks collectively enable cells to preserve functional integrity, recover from injury, and maintain homeostasis across fluctuating environmental and metabolic conditions. Cellular resilience is conceptualized as the dynamic capacity of biological systems to maintain or restore homeostasis under metabolic and environmental stress, a process in which adenosine signaling plays a central integrative role.

The regulatory effects of adenosine are organ- and context-specific. In the cardiovascular system, adenosine protects the myocardium from ischemic injury by promoting coronary vasodilation, reducing myocardial oxygen demand, and attenuating inflammatory responses [53]. In pulmonary tissues, adenosine signaling contributes to airway homeostasis, regulating inflammatory cell activation, epithelial repair, and fibroblast function [54,55]. However, chronic or dysregulated adenosine signaling may exacerbate pathological remodeling, airway hyperresponsiveness, and pulmonary fibrosis, highlighting the dual nature of this signaling system [56,57,58,59]. Within the central nervous system, adenosine functions as a neuromodulator, controlling neuronal excitability, sleep–wake cycles, synaptic plasticity, and neuroinflammation [60,61]. In the tumor microenvironment, hypoxia-driven adenosine accumulation suppresses cytotoxic T cell and natural killer (NK) cell activity, facilitating immune evasion and tumor progression [62,63,64]. These diverse effects underscore the complexity and context-dependence of adenosine-mediated regulation across biological systems.

Despite extensive research, critical gaps remain in our understanding of adenosine signaling. While individual receptor subtypes have been characterized in isolation, the spatiotemporal dynamics of receptor crosstalk, downstream signaling integration, and context-dependent effects remain incompletely defined. The mechanisms underlying receptor-specific responses in chronic versus acute stress, immune modulation, and tissue-specific fibrosis are not fully elucidated. Moreover, translational and therapeutic exploitation of adenosine signaling remains limited by challenges in selectively targeting receptor subtypes, avoiding off-target effects, and optimizing delivery strategies. Addressing these gaps is crucial for harnessing adenosine signaling in precision medicine and for designing next-generation therapeutic interventions.

Taken together, extracellular adenosine should be understood not only as a master integrator of metabolic and environmental stress signals but also as a dynamically regulated signaling network with direct translational relevance. By coordinating adaptive cellular responses across diverse physiological systems, adenosine signaling bridges fundamental mechanisms of stress adaptation with clinically meaningful outcomes. This dual role positions adenosine signaling as both a mechanistically robust regulatory framework and a therapeutically actionable target, offering significant opportunities for intervention across a broad spectrum of human diseases, including cardiovascular, inflammatory, neurological, and oncological conditions. By bridging foundational mechanistic biology with rapidly evolving translational therapeutics, this review aims to provide a systems-level conceptual framework for understanding adenosine signaling as both a master regulator of cellular stress adaptation and a clinically actionable therapeutic axis in human disease.

## 2. Literature Review Search Strategy

This study is defined as a structured narrative review rather than a fully systematic review, as it integrates mechanistic, translational, and clinical evidence without strict PRISMA-based inclusion criteria. A structured narrative review of adenosine signaling networks was conducted using the biomedical databases PubMed and Scopus, covering the period from January 2000 to February 2026 to capture both foundational discoveries and the latest advances in receptor biology, signaling pathways, and translational applications [65]. The search employed a combination of controlled vocabulary (MeSH/Emtree terms) and free-text keywords, using Boolean operators to integrate mechanistic, physiological, and clinical dimensions: (“adenosine signaling” OR “adenosine receptor” OR “A1 receptor” OR “A2A receptor” OR “A2B receptor” OR “A3 receptor” OR “CD39” OR “CD73”) AND (“cardiovascular disease” OR “ischemia-reperfusion” OR “pulmonary fibrosis” OR “asthma” OR “neurodegeneration” OR “tumor microenvironment” OR “cancer immunotherapy”) AND (“cAMP–PKA signaling” OR “MAPK pathway” OR “PI3K–Akt signaling” OR “HIF-1α” OR “hypoxia-inducible factor”). After retrieval, duplicate records were removed, and titles and abstracts were screened independently for relevance to adenosine-mediated physiological or pathological processes. Full-text articles of potentially eligible studies were assessed using predefined inclusion criteria: peer-reviewed research articles or authoritative reviews providing mechanistic, translational, or clinical insights into extracellular adenosine metabolism, receptor pharmacology, or therapeutic modulation of the CD39–CD73–adenosine axis. Exclusion criteria encompassed non-peer-reviewed reports, conference abstracts without full manuscripts, studies unrelated to adenosine signaling, and duplicate publications. Additional relevant studies were identified through manual screening of references in highly cited articles and recent comprehensive reviews. This structured yet flexible approach allowed for the integration of mechanistic, preclinical, and clinical evidence, ensuring that the review reflects the current state-of-the-art understanding of adenosine signaling across cardiovascular, pulmonary, neurological, and oncological contexts, while maintaining reproducibility and methodological transparency. To strengthen mechanistic rigor and translational relevance, priority was given to original experimental and clinical investigations whenever foundational receptor biology, intracellular signaling pathways, extracellular nucleotide metabolism, immunometabolic regulation, or therapeutic interventions were discussed. Review articles were selectively incorporated to provide conceptual integration, multidisciplinary synthesis, and contextual interpretation across highly heterogeneous biomedical domains. Following comprehensive reassessment of the reference architecture, approximately 60–65% of all cited references represent original experimental or clinical studies, while the remaining references consist of narrative reviews, systematic reviews, meta-analyses, and authoritative conceptual articles included to support integrative interpretation and translational continuity across multiple physiological systems and disease contexts.

## 3. Molecular Architecture of the Adenosine Signaling System

The biological influence of adenosine begins with its tightly regulated extracellular generation during metabolic stress [7]. In healthy tissues, extracellular ATP concentrations remain extremely low because nucleotides are rapidly hydrolyzed by ectonucleotidases expressed on endothelial cells, immune cells, and epithelial surfaces [12,66]. This enzymatic machinery ensures that nucleotide signaling remains spatially restricted and responsive to local tissue conditions. During cellular injury or metabolic stress, however, ATP release increases dramatically [67]. Hypoxia, mechanical strain, inflammatory signaling, and cell damage all promote ATP efflux into the extracellular space. Once released, ATP becomes the substrate for the CD39–CD73 enzymatic cascade that generates adenosine [14,68,69,70]. Extracellular nucleotide metabolism occurs through both canonical CD39–CD73 pathways and non-canonical pathways involving CD38/CD203a/CD73 as well as extracellular cAMP-to-adenosine conversion mechanisms, thereby contributing to dynamic adenosine generation. Beyond CD39 and CD73, additional ectonucleotidases including alkaline phosphatases, prostatic acid phosphatase (PAP), and nucleotide pyrophosphatase/phosphodiesterases (NPPs) contribute to extracellular nucleotide metabolism, each exhibiting distinct substrate specificity, tissue distribution, and regulatory mechanisms [38].

**Figure 1:**
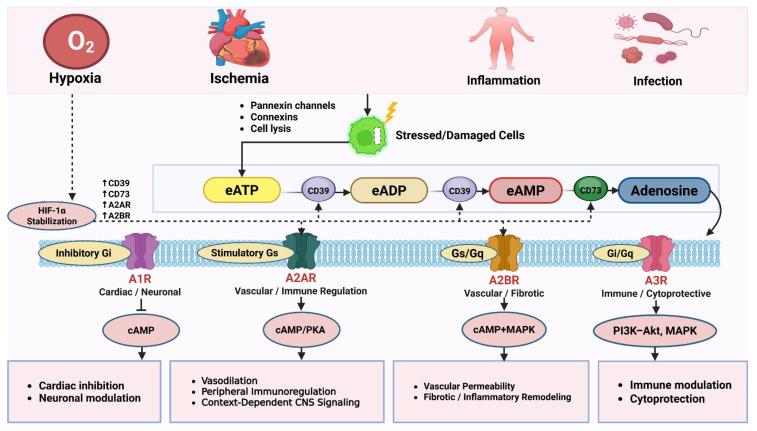
Adenosine signaling cascade during metabolic and environmental stress.

Adenosine is synthesized in the extracellular fluid as a highly conserved “defensive” response to changes in energy status of vital organs. It acts as a local paracrine humoral factor as well as a systemic factor in the blood. Equilibrative nucleoside transporters (ENTs) and concentrative nucleoside transporters (CNTs) regulate bidirectional adenosine flux across cellular membranes and play a central role in controlling extracellular adenosine availability and intracellular metabolism [71]. The biologic actions of adenosine are mediated by four GPCRs- A1, A2A, A2B, and A3, which have distinct affinities and efficacies, tissue, and subcellular distributions, as well as distinct physiological and pathological actions and signal transduction pathways. Adenosine receptors exhibit the ability to form homo- and heteromeric complexes, which significantly influence ligand affinity, receptor signaling bias, and downstream functional specificity. This receptor heteromerization represents a critical layer of regulation in purinergic signaling networks [72]. Receptor desensitization, internalization, and crosstalk with other GPCR systems further contribute to context-dependent signaling outcomes. Adenosine receptors are expressed across a wide range of cell types including endothelial, immune, epithelial, neuronal, and stromal cells. The A1 receptor is present in the heart, brain, and white adipose tissue. Its association with inhibitory Gi proteins reduces the adenylate cyclase activity and increases the K^+^ channel activity leading to a reduction in cyclic AMP [73,74,75]. A1 receptor agonists decrease heart rate and atrioventricular node transit time, thereby decreasing the myocardial oxygen demand. A1 receptors play critical roles in cardiac conduction, neuronal inhibition, and renal function. They inhibit neurotransmitter release and reduce neuronal excitability [76]. The A2A receptor is associated with stimulatory Gs proteins [77]. It activates adenylate cyclase and thereby increases cyclic AMP [78]. It activates protein kinase A and its various target phosphoproteins. It is also involved in gene expression and metabolism and therefore leads to vasodilation. It reduces the synthesis and release of proinflammatory cytokines and inhibits the immune and inflammatory actions of various cells [79]. The A2B receptor has a significantly lower affinity for adenosine and binds only to very high levels of this nucleoside achieved under pathophysiological conditions rather than under normal physiological conditions [80]. The receptor therefore produces its various cellular and physiological actions at high concentrations of extracellular adenosine achieved under pathophysiological conditions [81]. The A2B receptor couples to both Gs and Gq proteins, leading to activation of adenylate cyclase, phospholipase C signaling, and downstream MAPK pathways. It increases vascular permeability, induces fibroblast proliferation, and modulates inflammatory cytokine production [82,83]. The A3 receptor represents a versatile signaling node capable of activating both inhibitory and stimulatory pathways depending on the cellular environment [84]. The A3 receptors are associated with inhibitory Gi proteins in some cells and activate mitogen-activated protein kinases and phosphoinositide-3 kinase in other cells [85]. They can also modulate apoptosis and influence various aspects of immune modulation and thereby produce cytoprotective effects [86,87]. Metabolic stress triggers extracellular ATP release and its enzymatic conversion to adenosine, which activates adenosine receptor-mediated signaling pathways involved in immune regulation, inflammation, and cytoprotection (Figure 1).

Metabolic stress conditions such as hypoxia, ischemia, inflammation, and infection promote the release of extracellular ATP (eATP) from stressed or damaged cells via pannexin channels, connexins, or cell lysis. Extracellular ATP (eATP) and extracellular ADP (eADP are sequentially hydrolyzed by CD39 to AMP, which is further converted to adenosine by CD73. The generated extracellular adenosine activates four G-protein-coupled receptors: A1R (Gi), A2AR (Gs), A2BR (Gs/Gq), and A3R (Gi/Gq). These receptors regulate downstream signaling pathways including cAMP/PKA, MAPK, and PI3K–Akt, mediating cardiac and neuronal modulation, immune regulation, inflammatory responses, fibrotic signaling, and cytoprotection. Under hypoxic conditions, HIF-1α stabilization upregulates CD39/CD73 expression, enhancing extracellular adenosine generation and facilitating metabolic adaptation. HIF-1α also upregulates A2A and A2B receptor expression, further amplifying adenosine-mediated signaling under hypoxic conditions.

Abbreviations: eATP, extracellular adenosine triphosphate; eADP, extracellular adenosine diphosphate; AMP, adenosine monophosphate; GPCR, G protein-coupled receptor; cAMP, cyclic adenosine monophosphate; PKA, protein kinase A; MAPK, mitogen-activated protein kinase; PI3K–Akt, phosphoinositide 3-kinase–protein kinase B; HIF-1α, hypoxia-inducible factor-1 alpha.

Intracellularly, adenosine serves as a metabolic hub linking purine metabolism, redox balance, and energy sensing [88]. Adenosine deaminase (ADA) converts adenosine into inosine, contributing to purine degradation and generation of metabolites such as xanthine and uric acid, which possess redox-modulating properties [89]. Adenosine kinase (ADK) phosphorylates adenosine into AMP, linking adenosine metabolism to AMP-activated protein kinase (AMPK)-mediated regulation of cellular energy homeostasis [88]. Additionally, adenosine participates in transmethylation and transsulfuration pathways through interactions with homocysteine, thereby integrating into broader metabolic networks [90,91].

## 4. Integration of Adenosine Signaling with Major Intracellular Pathways

Although adenosine receptors belong to the G-protein-coupled receptor superfamily, their biological influence extends far beyond simple modulation of cyclic AMP levels. Adenosine receptor-induced signaling pathways are involved in the regulation of a variety of biological processes including gene expression, metabolism and cell survival. One of the important signaling pathways engaged by many adenosine receptor-induced cellular effects is the cAMP-protein kinase A (PKA) pathway [92]. Activation of A2A or A2B receptors increases adenylate cyclase activity and resulting cAMP levels. The increase in intracellular cAMP activates PKA that in turn catalyzes the phosphorylation of numerous target proteins including CREB and other transcription factors, thereby modulating the expression of genes involved in inflammation, metabolism and cell survival [93,94].

In parallel, adenosine receptor activation can stimulate the mitogen-activated protein kinase pathway [95]. MAPK signaling represents one of the most important regulatory networks controlling cell proliferation, differentiation, and stress responses. Activation of the MAPK cascades by A2B and A3 receptor subtypes regulates fibroblast activation, pro-inflammatory cytokine production, and tissue repair processes [46,96]. In parallel, A2A and A2B receptor-mediated activation of the PI3K–Akt signaling pathway plays a critical role in promoting cell survival under conditions of energy stress [97]. This pathway further modulates cellular metabolism and confers protection against apoptosis, thereby enhancing tissue resilience during metabolic stress [98]. The PI3K–Akt signaling pathway is involved in various cellular processes including glucose metabolism, protein synthesis and growth [99]. The regulation of adenosine signaling by various cellular stresses is multifaceted. Hypoxia further amplifies adenosine signaling through the activity of the transcription factor HIF-1α [100]. Under low oxygen conditions, stabilization of HIF-1α increases expression of CD39 and CD73 enzymes, thereby increasing the extracellular level of adenosine [14,101]. In addition, HIF-1α increases the expression of A2B receptors thereby enhancing the cellular response to adenosine [36,102,103]. Thus, hypoxia increases the adenosine signaling in a feed-forward manner. The ability of adenosine to interact with a variety of signaling pathways such as cAMP, MAPK, PI3K–Akt and HIF-1α provides an insight into the diverse roles of this molecule in cellular processes including inflammation, metabolism, and repair and immune regulation. Adenosine signaling exerts system-wide effects through receptor-specific activation of intracellular pathways, integrating physiological regulation with pathological remodeling across multiple organ systems, as summarized in Table 1.

## 5. Adenosine Signaling in Cardiovascular Protection and Ischemic Adaptation

The cardiovascular system represents one of the earliest and most extensively studied contexts in which adenosine signaling exerts profound physiological effects. The heart is an extremely energetic organ which requires a large amount of ATP for its operation. Due to the high metabolic demands, even transient decreases in oxygen availability can rapidly compromise energy homeostasis, triggering adaptive responses that are critically mediated by extracellular adenosine. Myocardial ischemia results in a rapid decrease in intracellular ATP with a corresponding increase in the extracellular concentration of adenosine [146]. This surge in adenosine functions as a central protective signal, modulating vascular, metabolic, and cellular processes to preserve myocardial integrity [147]. The elevation of adenosine in the coronary vasculature induces receptor-specific vasodilation, thereby enhancing blood flow to ischemic myocardium [148,149]. Vascular smooth muscle A2 receptor activation increases cyclic AMP production, which promotes relaxation and dilation of coronary vessels, ensuring oxygen and nutrient delivery to stressed cardiac tissue [150]. Concurrently, adenosine engagement of cardiac A1 receptors reduces heart rate and contractility, conserving high-energy phosphates and minimizing ischemic injury [30,53].

Adenosine is also a critical mediator of ischemic preconditioning [151], wherein brief, sublethal ischemic episodes activate endogenous defense pathways that reduce injury from subsequent prolonged ischemia [152]. Mechanistically, adenosine receptor activation during preconditioning stimulates protein kinase C (PKC) signaling and enhances mitochondrial protective mechanisms, including the opening of mitochondrial ATP-sensitive potassium channels, upregulation of anti-apoptotic proteins, and reduction of reactive oxygen species [153,154]. Translational studies have explored exogenous adenosine administration during acute myocardial infarction and percutaneous coronary intervention (PCI), aiming to mitigate reperfusion injury [155,156]. Adenosine confers cardioprotection by attenuating neutrophil activation, limiting oxidative stress, preserving endothelial barrier integrity, and modulating inflammatory cascades [157,158]. These multifaceted mechanisms highlight adenosine as a master integrator of cardiovascular stress adaptation, linking metabolic cues to systemic and cellular protective responses.

## 6. Adenosine Signaling in Pulmonary Inflammation and Fibrotic Remodeling

The lung represents a complex interface between the internal and external environment, constantly exposed to aerosolized particles, pathogens, allergens, and gases. Adenosine signaling constitutes a pivotal homeostatic mechanism in the lung, coordinating immunological surveillance, barrier function, and adaptive responses to environmental and metabolic stress [159]. Under physiological conditions, extracellular adenosine concentrations remain low [154]. However, during acute or chronic lung injury, extracellular ATP accumulates due to epithelial cell damage and release from activated immune cells [70]. Subsequent enzymatic conversion by ectonucleotidases such as CD39 and CD73 generates high local concentrations of adenosine, modulating immune and tissue responses [160,161].

Adenosine exerts protective anti-inflammatory effects during acute lung injury, primarily through A2A receptor activation on neutrophils and macrophages, reducing pro-inflammatory cytokine release and limiting tissue damage [79,162]. This underscores its critical role in conditions such as acute respiratory distress syndrome (ARDS) and sepsis-associated lung injury, where rapid modulation of inflammatory responses can be lifesaving. In chronic pulmonary disease, the role of adenosine is more complex. Prolonged elevation of extracellular adenosine contributes to fibrotic remodeling, airway hyperresponsiveness, and lung dysfunction [56,163,164,165]. High adenosine levels activate A2B receptors on fibroblasts, epithelial cells, and vascular endothelium, triggering intracellular signaling cascades, including MAPK pathways, which drive collagen synthesis, fibroblast proliferation, and cytokine production [154,166]. These processes promote excessive extracellular matrix deposition, culminating in structural lung alterations and impaired gas exchange [167,168].

Hypoxia acts as a key amplifier of this process, stabilizing hypoxia-inducible factor-1α (HIF-1α), which enhances expression of CD39, CD73, and A2B receptors, creating a feed-forward loop that couples metabolic stress to fibrotic remodeling [116,159,169,170]. CD39, a transmembrane ectonucleoside triphosphate diphosphohydrolase, hydrolyzes extracellular ATP and ADP to AMP, while CD73, a GPI-anchored 5′-nucleotidase, converts AMP to adenosine, thereby amplifying extracellular adenosine generation [14,101]. This hypoxia–adenosine axis integrates metabolic stress with structural and functional remodeling, highlighting an adaptive yet potentially maladaptive response in chronic lung disease. Recent evidence also implicates adenosine in chronic respiratory diseases such as asthma and chronic obstructive pulmonary disease (COPD), potentially via airway hyperreactivity, mast cell degranulation, and mediator release [171,172,173]. For instance, inhaled adenosine can provoke bronchospasm in sensitive asthmatic patients, demonstrating the dualistic role of adenosine in balancing acute protection with chronic structural and inflammatory pathology [174,175]. Therapeutically, selective receptor targeting is being explored. A2B receptor antagonism may inhibit fibroblast activation and limit fibrotic remodeling while preserving the anti-inflammatory benefits mediated through A2A receptors [139,176]. These interventions exemplify a broader principle in adenosine biology that the physiological and pathological consequences of adenosine are governed not only by receptor subtype specificity but also by the spatial and temporal dynamics of adenosine production within tissue microenvironments. Emerging evidence suggests context-dependent dual roles of adenosine signaling, where protective anti-inflammatory effects may paradoxically promote chronic disease progression, highlighting the need for receptor-specific therapeutic targeting [177,178].

## 7. Neuromodulatory Functions of Adenosine in the Central Nervous System

The brain is a highly metabolic organ and uses approximately 20% of the oxygen and glucose that are consumed by the whole body, despite accounting for only a small fraction of body mass [179]. Therefore, precise regulation of neuronal activity is essential for maintaining energy homeostasis and for preventing damage to neurons due to excitotoxicity. Adenosine has been implicated as a key neuromodulator involved in the regulation of brain activity and energy metabolism [1,180]. Unlike conventional neurotransmitters that are stored within synaptic vesicles, adenosine is synthesized as an ATP catabolite in the extracellular space of the synapse and therefore signals a response to increased energy demand of active neurons [181,182].

Adenosine receptors are widely distributed throughout the central nervous system, including the cortex, hippocampus, thalamus, basal ganglia, and cerebellum, with expression in neurons, astrocytes, microglia, and synaptic terminals. The neuromodulatory effects of adenosine in the brain are mediated by the A1 receptor [94,112,183]. These receptors are found throughout the cerebral cortex, hippocampus and thalamus [184,185]. When the A1 receptors are stimulated by adenosine it results in a decrease in adenylate cyclase activity. This leads to a decrease in intracellular cyclic AMP (cAMP) which in turn leads to a decrease in neurotransmitter release from the presynaptic terminal [73,186]. Overall, adenosine acts to decrease excitatory neurotransmission and acts to stabilize neuronal activity [183,187]. One of the most familiar physiological consequences of this signaling system is the regulation of sleep–wake homeostasis [60,188,189]. During prolonged wakefulness, metabolic activity in the brain leads to progressive accumulation of extracellular adenosine. This increase suppresses activity within arousal-promoting neural circuits and contributes to the sensation of sleep pressure that develops over the course of the day [190]. The widespread consumption of caffeine illustrates the importance of this neuromodulatory pathway [191]. Caffeine is a reversible non-selective antagonist to the A1 and A2A receptors. As an antagonist to the adenosine receptors, it inhibits the sleep promoting effects of adenosine and therefore results in wakefulness and alertness [192,193]. The A1 receptors also play a role in neuroprotection in the brain [194]. Following ischemic stroke or traumatic brain injury there is an overabundance of glutamate in the synaptic cleft which can cause excitotoxicity in the neurons [195,196,197]. The A1 receptors help to decrease glutamate release and decrease the depolarization of the neuronal membranes, therefore preventing further excitotoxicity [94,198].

Adenosine signaling has also emerged as an important modulator of neuroinflammation [112,199]. The resident immune cells of the brain, microglia, express nearly all known adenosine receptor subtypes [199,200]. Activation of A2A receptors decreases the production of pro-inflammatory cytokines and subsequent neuronal damage associated with chronic neuroinflammation [201,202]. These mechanisms have important implications for neurodegenerative diseases such as Parkinson’s disease (PD) and Alzheimer’s disease (AD) [203,204]. In the basal ganglia, A2A receptors modulate dopaminergic function in neural circuits involved in motor control and an A2A receptor antagonist has been clinically tested to assess its ability to improve motor function in PD patients [203,205]. Emerging evidence also suggests that adenosine signaling may influence processes such as synaptic plasticity, learning, and memory formation [112,206,207]. Through interactions with MAPK and PI3K–Akt signaling networks, adenosine receptors can regulate gene transcription programs that influence long-term neuronal adaptation [206,208]. Thus, within the brain, adenosine functions as far more than a metabolic byproduct. It operates as a sophisticated neuromodulatory signal that integrates neuronal activity, metabolic demand, and immune signaling to maintain neural circuit stability.

## 8. Adenosine Metabolism and Immune Suppression in the Tumor Microenvironment

Cancer immunology represents one of the most rapidly advancing areas in adenosine biology. Tumors actively exploit metabolic signaling networks to remodel the tumor microenvironment (TME) and evade immune surveillance. Among these mechanisms, the adenosine signaling axis has emerged as a central regulator of tumor-associated immunosuppression, integrating metabolic stress cues with immune evasion mechanisms [96,118]. Solid tumors frequently experience hypoxia due to rapid proliferation outpacing neovascularization, resulting in stabilization of hypoxia-inducible factor 1-alpha (HIF-1α). HIF-1α functions as a master transcriptional regulator, upregulating genes involved in angiogenesis, glycolysis, nucleotide metabolism, and survival pathways [209,210,211]. Importantly, HIF-1α induces expression of CD39 and CD73 on tumor and stromal cells, thereby enhancing the enzymatic conversion of extracellular ATP and ADP into AMP and adenosine, resulting in elevated local concentrations of extracellular adenosine [14,50,101,116]. Simultaneously, HIF-1α promotes upregulation of A2A and A2B adenosine receptors on immune and tumor cells, creating a spatiotemporally coordinated immunosuppressive adenosine gradient within the TME [212,213].

Cytotoxic T lymphocytes (CTLs) and natural killer (NK) cells, the principal effector populations mediating tumor immune surveillance, are highly susceptible to adenosine-mediated suppression [36,214]. A2A receptor engagement on CTLs increases intracellular cAMP, activating PKA and downstream exchange proteins directly activated by cAMP (EPAC: Exchange Protein Activated by cAMP), which inhibit TCR (T cell receptor)-proximal signaling, reduce nuclear factor of activated T cell (NFAT) nuclear translocation, and suppress cytokine production (e.g., IFN-γ: Interferon-gamma, TNF-α: Tumor Necrosis Factor-alpha), collectively dampening cytotoxic function [215]. Similarly, A2B receptor signaling in NK cells impairs cytotoxic granule exocytosis, IFN-γ secretion, and tumor-targeted lytic activity [216]. Adenosine also modulates the suppressive arm of the immune system. Regulatory T cells (Tregs) within the TME express high levels of CD39/CD73, allowing them to convert ATP released by dying or stressed cells into adenosine, autonomously reinforcing local immunosuppression [36,118,217]. Adenosine promotes Treg differentiation, enhances expression of immunosuppressive molecules CTLA-4 (Cytotoxic T lymphocyte-associated protein 4), PD-1 (Programmed Cell Death Protein 1) and stimulates secretion of IL-10 (Interleukin-10) and TGF-β (Transforming Growth Factor-beta), creating a feed-forward loop that limits effector T cell and NK cell activity [218]. In addition, myeloid-derived suppressor cells (MDSCs) and tumor-associated macrophages (TAMs) are polarized toward pro-tumor phenotypes under high-adenosine concentrations, with enhanced arginase-1 activity, IL-10 secretion, and reduced antigen-presenting capacity, further consolidating immune evasion [219]. Recent advances have demonstrated that adenosine-mediated immunosuppression extends beyond T cells and NK cells to include modulation of dendritic cell maturation, macrophage polarization, and metabolic reprogramming within the tumor microenvironment, highlighting its central role in tumor immune escape [64,118,212,213].

The metabolic architecture of the TME (tumor microenvironment) thus forms an integrated network where hypoxia, nucleotide metabolism, and immune regulation converge. Extracellular adenosine acts as a metabolic–immune nexus, translating stress signals into immunosuppressive signaling, suppressing cytotoxicity, and facilitating tumor survival and expansion. Notably, this axis also intersects with other oncogenic and immunoregulatory pathways, including PD-1/PD-L1 (Programmed Cell Death Protein 1/Programmed Cell Death-Ligand 1), CTLA-4, and TGF-β signaling, highlighting role of adenosine as a central node integrating metabolic stress and immune checkpoint pathways [118]. These mechanistic insights have catalyzed therapeutic strategies targeting the CD39–CD73–adenosine axis, with translational and clinical implications. Pharmacological inhibition of CD39 or CD73 disrupts adenosine production [14], while selective antagonism of A2A or A2B receptors restores CTL and NK cell function, diminishes Treg-mediated suppression, and reprograms MDSCs and TAMs toward anti-tumor phenotypes [220,221,222]. Preclinical studies demonstrate synergistic effects when adenosine pathway inhibitors are combined with immune checkpoint inhibitors, adoptive T cell therapies, or cancer vaccines, enhancing tumor regression and survival outcomes [223,224]. Furthermore, strategies targeting adenosine transporters (ENTs) or adenosine-metabolizing enzymes provide complementary approaches to modulate the extracellular adenosine pool, potentially enhancing immune-mediated tumor clearance [212].

Overall, adenosine constitutes a central metabolic–immune hub within the TME, translating hypoxia and metabolic stress into multifaceted immune suppression. By modulating extracellular nucleotide metabolism, receptor-mediated signaling, and intracellular immune effector pathways, adenosine orchestrates tumor adaptation and progression while offering multiple targets for therapeutic intervention. Understanding this intricate network provides a foundation for the development of precision immunotherapies that selectively restore anti-tumor immunity without compromising physiological homeostasis. Importantly, the role of adenosine signaling exhibits significant tissue-specific variability, necessitating a balanced interpretation across organ systems. While oncology-focused evidence is robust, emerging data from CNS and pulmonary studies highlight equally critical roles in neurodegeneration and chronic lung disease, thereby reinforcing the need for integrative, system-wide therapeutic strategies. The translational relevance of adenosine signaling across multiple disease contexts, including oncology, cardiovascular disorders, neurodegeneration, and pulmonary diseases, is summarized in Table 2. This table integrates receptor subtype specificity with therapeutic strategies, clinical development stages, mechanistic insights, and current limitations of adenosine-targeted interventions.

Adenosine signaling should not be interpreted merely as an isolated receptor-mediated pathway, but rather as a dynamic systems-level adaptive network integrating metabolic stress sensing, hypoxic adaptation, immune regulation, mitochondrial homeostasis, and tissue resilience. This broader conceptual perspective may help explain the remarkable context-dependent duality of adenosine signaling, wherein protective cytoprotective effects observed during acute tissue injury may transition toward maladaptive immunosuppressive or profibrotic consequences under chronic pathological conditions. Such mechanistic complexity underscores the importance of tissue-specific, receptor subtype-selective, and temporally regulated therapeutic strategies targeting adenosine signaling pathways.

The expanding therapeutic landscape surrounding adenosine signaling highlights its growing importance within precision medicine and translational pharmacology. Emerging strategies targeting adenosine receptors, CD39/CD73-mediated extracellular nucleotide metabolism, equilibrative nucleoside transporters, and immunometabolic signaling pathways may offer novel opportunities for disease-specific therapeutic modulation across cardiovascular, inflammatory, neurodegenerative, pulmonary, and oncological disorders. However, future translational success will likely depend upon achieving greater receptor subtype selectivity, minimizing off-target systemic effects, and improving mechanistic understanding of context-dependent signaling dynamics within distinct tissue microenvironments.

## 9. Strengths and Limitations

The present review possesses several important strengths. Unlike many previously published reviews that focus predominantly on isolated adenosine receptor subtypes or single-organ disease mechanisms, this work provides a comprehensive systems-level synthesis positioning adenosine signaling as a central integrative network coordinating metabolic stress sensing, hypoxia-responsive adaptation, immune modulation, mitochondrial homeostasis, inflammatory regulation, and tissue resilience across multiple physiological and pathological conditions. A major strength of this review lies in its multidisciplinary integration of mechanistic receptor biology, extracellular nucleotide metabolism, purinergic signaling dynamics, immunometabolic regulation, and translational therapeutics within a unified conceptual framework spanning cardiovascular diseases, pulmonary disorders, neurodegeneration, inflammatory diseases, fibrosis, and oncology. Furthermore, substantial emphasis was placed on the incorporation of original experimental and translational studies to strengthen mechanistic interpretation and clinical relevance. The review also integrates several highly contemporary developments, including CD39/CD73-targeted immunotherapeutic strategies, adenosine-mediated tumor immune evasion, receptor subtype-selective pharmacology, equilibrative nucleoside transporter modulation, and emerging precision medicine perspectives relevant to translational pharmacology. Collectively, these features enhance the translational significance, conceptual depth, and multidisciplinary relevance of the review for both basic scientists and clinical investigators.

Nevertheless, certain limitations should also be acknowledged. First, although a structured literature search strategy was employed using major biomedical databases, the present work was conducted as a structured narrative review rather than a fully systematic review; therefore, PRISMA-guided study selection, formal quantitative synthesis, and structured risk-of-bias assessment were not performed. Second, due to the rapidly evolving and highly heterogeneous nature of adenosine biology, variability exists across experimental models, receptor subtype-specific effects, tissue microenvironments, and disease-specific signaling mechanisms, which may limit direct translational generalizability. Third, despite prioritizing original experimental and clinical investigations wherever possible, selected review articles were intentionally incorporated to facilitate multidisciplinary conceptual integration and provide broader interpretive continuity across diverse biomedical domains. Finally, although adenosine signaling represents a highly promising therapeutic target, several translational challenges remain unresolved, including receptor crosstalk complexity, context-dependent dual biological effects, pharmacodynamic heterogeneity, tissue-specific variability, and the current lack of sufficient long-term clinical validation for several emerging adenosine-targeted therapeutic strategies. These limitations underscore the need for further mechanistic, translational, and large-scale clinical investigations to refine therapeutic targeting approaches and improve disease-specific precision interventions involving adenosine signaling pathways.

## 10. Conclusions

Adenosine signaling has emerged as a central integrative network that orchestrates cellular responses to metabolic, hypoxic, and inflammatory stress across multiple physiological systems. Through tightly regulated extracellular nucleotide metabolism and receptor-mediated signaling via A1, A2A, A2B, and A3 receptor subtypes, adenosine dynamically modulates intracellular pathways involved in energy homeostasis, immune regulation, tissue protection, and cellular survival. These mechanistic insights firmly establish adenosine as a master regulator of cellular stress adaptation and homeostatic balance. Importantly, accumulating evidence now extends this mechanistic framework into the translational domain, positioning adenosine signaling as a clinically actionable therapeutic target. Dysregulation of adenosine pathways contributes to the pathophysiology of a wide spectrum of diseases, including cardiovascular disorders, chronic inflammatory conditions, neurodegenerative diseases, and cancer. Targeting specific components of the adenosine signaling axis, particularly receptor subtypes and ectonucleotidases, has demonstrated promising therapeutic potential in preclinical and clinical settings. Bridging fundamental systems biology with translational application, adenosine signaling represents a uniquely positioned regulatory network that integrates environmental stress sensing with adaptive cellular and tissue-level responses. Future research should focus on refining receptor subtype-specific interventions, improving spatiotemporal targeting strategies, and advancing precision medicine approaches to fully harness the therapeutic potential of this pathway. Collectively, adenosine signaling should be viewed not only as a mechanistic cornerstone of cellular stress adaptation but also as a strategically exploitable target for innovative therapeutic development across diverse human diseases.

## Figures and Tables

**Table 1:** Tissue-specific adenosine receptor functions and pathways.

Domain/Tissue	Primary Receptor(s)	Intracellular Pathways Activated	Physiological/Protective Effects	Pathological Effects	Translational Insights
Cardiovascular System [32,34]	A1, A2A	cAMP–PKA, MAPK, PI3K–Akt	Coronary vasodilation, reduced heart rate, ischemic preconditioning, myocardial protection [104,105]	Impaired oxygen delivery during chronic ischemia may lead to maladaptive remodeling [106]	Adenosine infusion during acute MI, A1 receptor agonists, preconditioning mimetics [107]
Pulmonary Tissues [34,56]	A2A, A2B, A3	cAMP–PKA, MAPK, PI3K–Akt, HIF-1α	Anti-inflammatory effects, epithelial repair, modulation of neutrophils and macrophages [108]	Chronic A2B activation → fibroblast proliferation, airway remodeling, pulmonary fibrosis, asthma exacerbation [109]	A2B receptor antagonists, adenosine analogs for acute lung injury [110]
Central Nervous System [111,112]	A1, A2A	cAMP–PKA, MAPK, PI3K–Akt	Neuromodulation, sleep–wake regulation, neuroprotection, inhibition of excitotoxicity [113,114]	Dysregulated signaling may contribute to neurodegenerative disease, impaired cognition [115]	A2A receptor antagonists in Parkinson’s disease, neuroprotective strategies in stroke [115]
Tumor Microenvironment [36,116]	A2A, A2B, A3	cAMP–PKA, PI3K–Akt, HIF-1α	Immune suppression of T cells and NK cells, promotion of Treg expansion [117]	Facilitate tumor immune escape, angiogenesis, metabolic adaptation [118]	CD39/CD73 inhibitors, receptor-specific antagonists, combination with checkpoint inhibitors [119]
Metabolic/Hypoxic Tissues [34,120]	A2B, A3	HIF-1α, MAPK, PI3K–Akt	Adaptive responses to hypoxia, ATP conservation, angiogenesis [121]	Chronic hypoxia → tissue fibrosis, metabolic dysregulation [122]	Modulation of HIF-1α–adenosine axis to prevent fibrosis and ischemic injury [123]
Immune Cells [36,37,124]	A2A, A2B, A3	cAMP–PKA, PI3K–Akt	Anti-inflammatory cytokine regulation, inhibition of overactive immune responses [96]	Immunosuppression during chronic inflammation or tumor progression [125]	Adenosine receptor antagonists to enhance immune responses in cancer [36]
Digestive System [126,127,128]	A2A, A2B	cAMP–PKA, MAPK, epithelial barrier signaling pathways	Maintenance of intestinal barrier integrity, regulation of motility, anti-inflammatory effects [129]	Inflammatory bowel disease, colorectal carcinogenesis, epithelial dysfunction [130]	A2A agonists for mucosal protection, A2B antagonists for inflammation control [131]
Reproductive System [132,133,134]	A1, A2A, A2B	cAMP–PKA, Ca^2+^ signaling, steroidogenic pathways	Regulation of spermatogenesis, ovarian function, uterine contractility [135]	Infertility, impaired gametogenesis, inflammatory reproductive disorders [135]	Emerging targets in fertility modulation and reproductive inflammatory diseases [136]
Renal System [137,138,139]	A1, A2A	cAMP–PKA, tubuloglomerular feedback signaling	Regulation of glomerular filtration rate, sodium reabsorption, renal protection [140]	Chronic kidney disease progression, fibrosis, altered hemodynamics [139]	A1 receptor antagonists in renal dysfunction and diuretic resistance [141]
Skeletal Muscle [142,143]	A1, A2A	AMPK activation, mitochondrial signaling pathways	Energy metabolism regulation, fatigue resistance, improved perfusion [142]	Muscle wasting, metabolic dysfunction [144]	Potential targets for metabolic and exercise-related disorders [145]

**Table 2:** Translational and therapeutic targeting of adenosine signaling across diseases.

Target	Disease Context	Therapeutic Strategy	Representative Drug	Mechanism	Key Limitations
A2A receptor [225,226,227,228]	Cancer (NSCLC, melanoma)	Antagonist	CPI-444 (Ciforadenant)	Enhances anti-tumor T cell response	Tumor heterogeneity, resistance mechanisms
A2A receptor [229,230,231]	Parkinson disease	Antagonist	Istradefylline	Modulates dopaminergic signaling	Limited efficacy in advanced stages
A1 receptor [76,232,233,234]	Cardiac arrhythmias, heart failure	Agonist	Adenosine	AV nodal conduction inhibition, inhibition of adenylate cyclase activity, modulation of protein kinase C, and opening of ATP-sensitive potassium channels	Short half-life, transient effects
CD73 (NT5E) [235,236,237,238]	Cancer immunotherapy	Inhibitor (mAb)	Oleclumab	Reduces adenosine-mediated immunosuppression	Compensatory pathways
CD39 (ENTPD1) [239,240]	Tumor microenvironment	Inhibitor	IPH5201	Blocks ATP degradation to adenosine	Incomplete pathway inhibition
A2B receptor [241,242,243]	Pulmonary fibrosis	Antagonist	GS-6201	Reduces fibroblast activation	Limited human data
ENT1 transporter [244,245]	Ischemia–reperfusion injury	Inhibitor	Dipyridamole	Increases extracellular adenosine levels	Non-specific effects

## Data Availability

No new data were created or analyzed in this study. Data sharing is not applicable to this article.

## Abbreviations

The following abbreviations are used in this manuscript:

ATP	Adenosine Triphosphate
ADP	Adenosine Diphosphate
AMP	Adenosine Monophosphate
ADA	Adenosine Deaminase
ADK	Adenosine Kinase
AMPK	AMP-Activated Protein Kinase
A1	Adenosine A1 Receptor
A2A	Adenosine A2A Receptor
A2B	Adenosine A2B Receptor
A3	Adenosine A3 Receptor
GPCR	G Protein-Coupled Receptor
MAPK	Mitogen-Activated Protein Kinase
PI3K–Akt	Phosphoinositide 3-Kinase–Protein Kinase B Pathway
cAMP	Cyclic Adenosine Monophosphate
PKA	Protein Kinase A
CREB	cAMP Response Element-Binding Protein
HIF-1α	Hypoxia-Inducible Factor-1 Alpha
CD39	Cluster of Differentiation 39 (ENTPD1; Ectonucleoside Triphosphate Diphosphohydrolase-1)
CD73	Cluster of Differentiation 73 (NT5E; Ecto-5′-Nucleotidase)
ENT	Equilibrative Nucleoside Transporter
CNT	Concentrative Nucleoside Transporter
NK	Natural Killer Cells
TME	Tumor Microenvironment
TCR	T Cell Receptor
CTL	Cytotoxic T Lymphocyte
NF-κB	Nuclear Factor Kappa B
ERK	Extracellular Signal-Regulated Kinase
CNS	Central Nervous System
COPD	Chronic Obstructive Pulmonary Disease
MI	Myocardial Infarction
AV node	Atrioventricular Node
NSCLC	Non-Small Cell Lung Cancer
ECM	Extracellular Matrix
Tregs	Regulatory T cells
